# Cronkhite-Canada syndrome: An atypical presentation with subungual hyperkeratosis

**DOI:** 10.1016/j.jdcr.2023.12.005

**Published:** 2024-01-09

**Authors:** Peerada Sermswan, Pravit Asawanonda, Nopadon Noppakun, Chanat Kumtornrut

**Affiliations:** Division of Dermatology, Department of Medicine, Faculty of Medicine, Chulalongkorn University, King Chulalongkorn Memorial Hospital, The Thai Red Cross Society, Bangkok, Thailand

**Keywords:** acquired gastrointestinal polyposis, acrofacial lentigines, Cronkhite-Canada syndrome, juvenile hamartoma, nonhereditary polyposis, onychodystrophy

## Introduction

Cronkhite-Canada syndrome (CCS) is a rare disease described in the literature for almost a century; however, its pathogenesis remains unclear.^1^ Genetic predisposition, *Helicobactor pylori* infection and autoimmune processes has been described as the contributing factors.^2^, ^3^, ^4^ It is characterized by gastrointestinal polyposis and a triad of ectodermal signs consisting of brownish macules on the acrofacial area, onychodystrophy, and nonscarring diffuse alopecia. The polyposis causes diarrhea and malabsorption, resulting in nutritional deficiencies, hypoalbuminemia, and electrolyte imbalance.^5^

Classic nail changes in CCS are onychodystrophy or nail brittleness.^6^^,^^7^ Studies have described the nail finding as thinning and splitting of nail plates or occasionally as onychomadesis.^5^ These characteristic nail findings were secondary to nutritional deficiencies, such as vitamin A deficiency. However, the former belief is challenged by the evidence of inflammation in a nail matrix biopsy.^8^ The precise pathophysiology underlying CCS changes and associated nail manifestations continue to elude our understanding. As a result, the diagnosis of CCS with unusual nail findings is challenging. Here, we present a patient with CCS with a unique subungual hyperkeratosis, a feature that has never been described elsewhere in the literature, along with onychodystrophy.

## Case report

**Fig 1:**
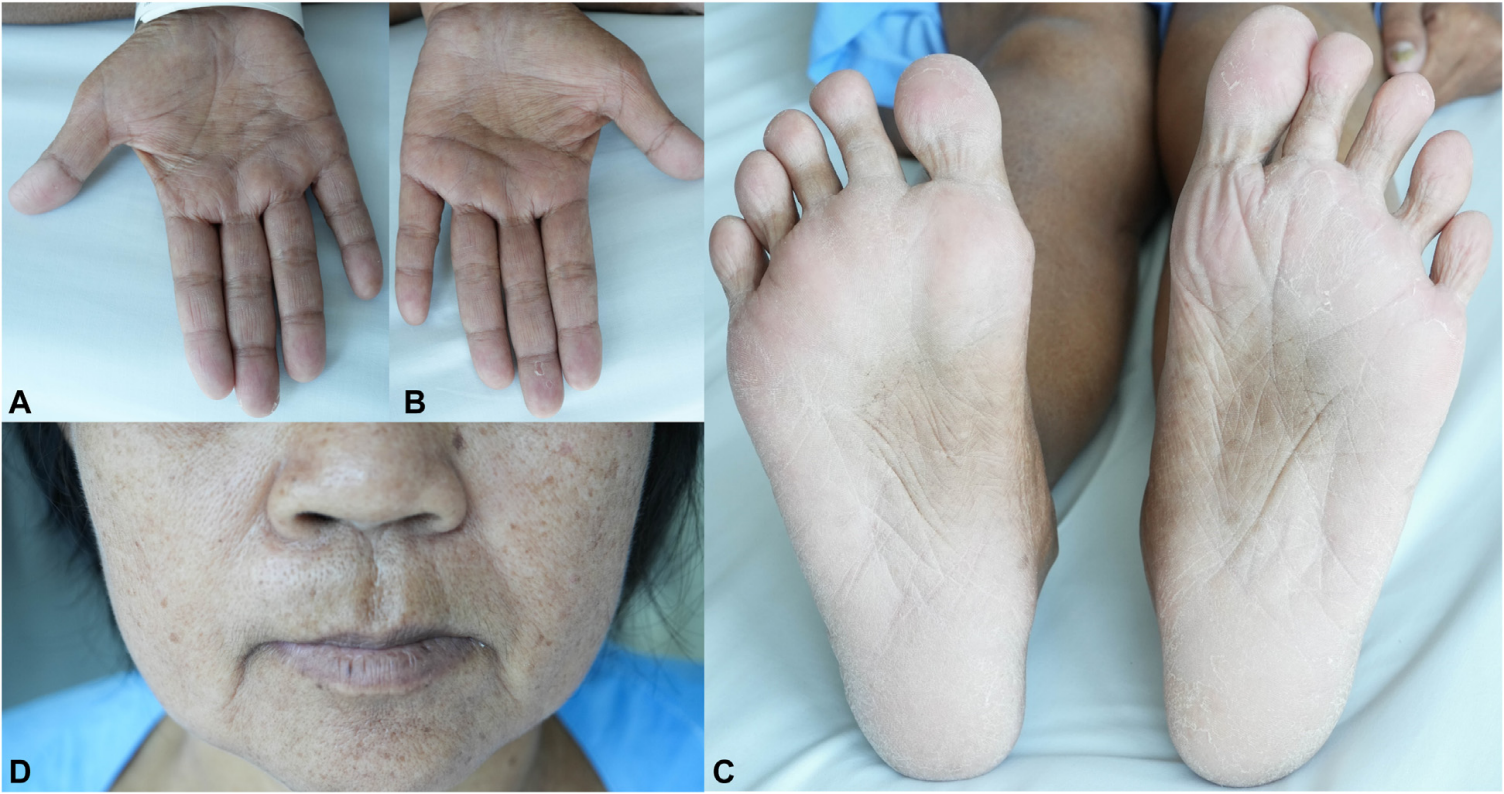
Cutaneous features of Cronkhite-Canada syndrome. Multiple *brownishmacules* on the palms (**A**, **B**), perioral area (**C**), and soles (**D**).

**Fig 2:**
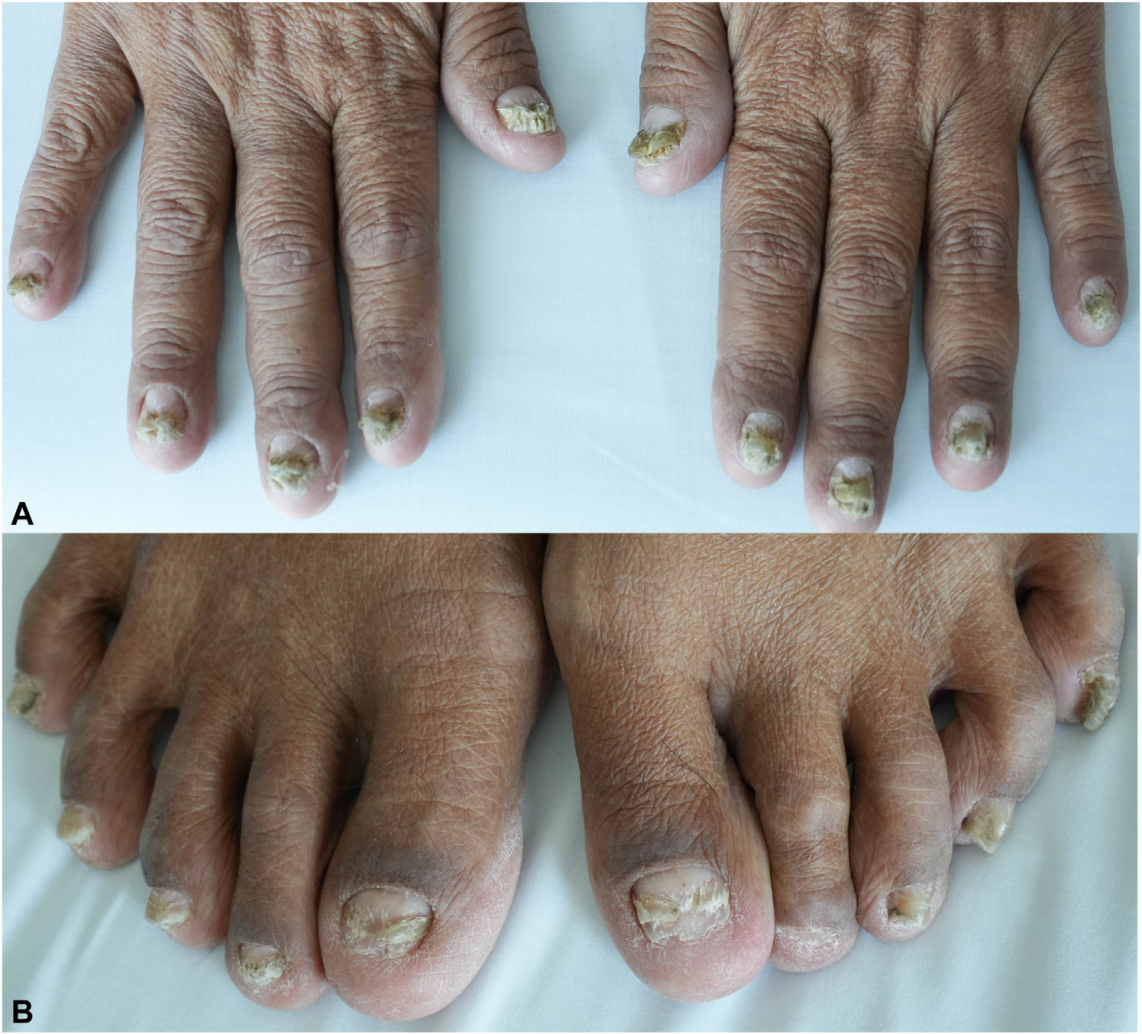
Nail features of Cronkhite-Canada syndrome. Marked subungual hyperkeratosis and onychorrhexis of fingernails (**A**) and toenails (**B**).

A 51-year-old Thai woman presented with hyperpigmented patches on her palm and soles for 4 years. She noticed thickening of her nails and progressive hair shedding. A few months later, chronic watery diarrhea arose, dysgeusia, and significant weight loss. Physical examination revealed lentigo-like brownish macules and patches on her palm, soles, lips, and perioral area (Fig 1, *A*-*D*). All nails revealed marked subungual hyperkeratosis and onychorrhexis (Fig 2, *A* and *B*). Diffuse nonscarring alopecia with a positive hair-pulling test was noted. No eyebrows, eyelashes, or axillary/pubic hair loss was reported. She denied having any underlying diseases, concurrent medications, allergies, previous surgical procedures, or history of arsenical exposure. She has normal eating habits. No family member had experienced gastrointestinal polyposis or nail disorders.

**Fig 3:**
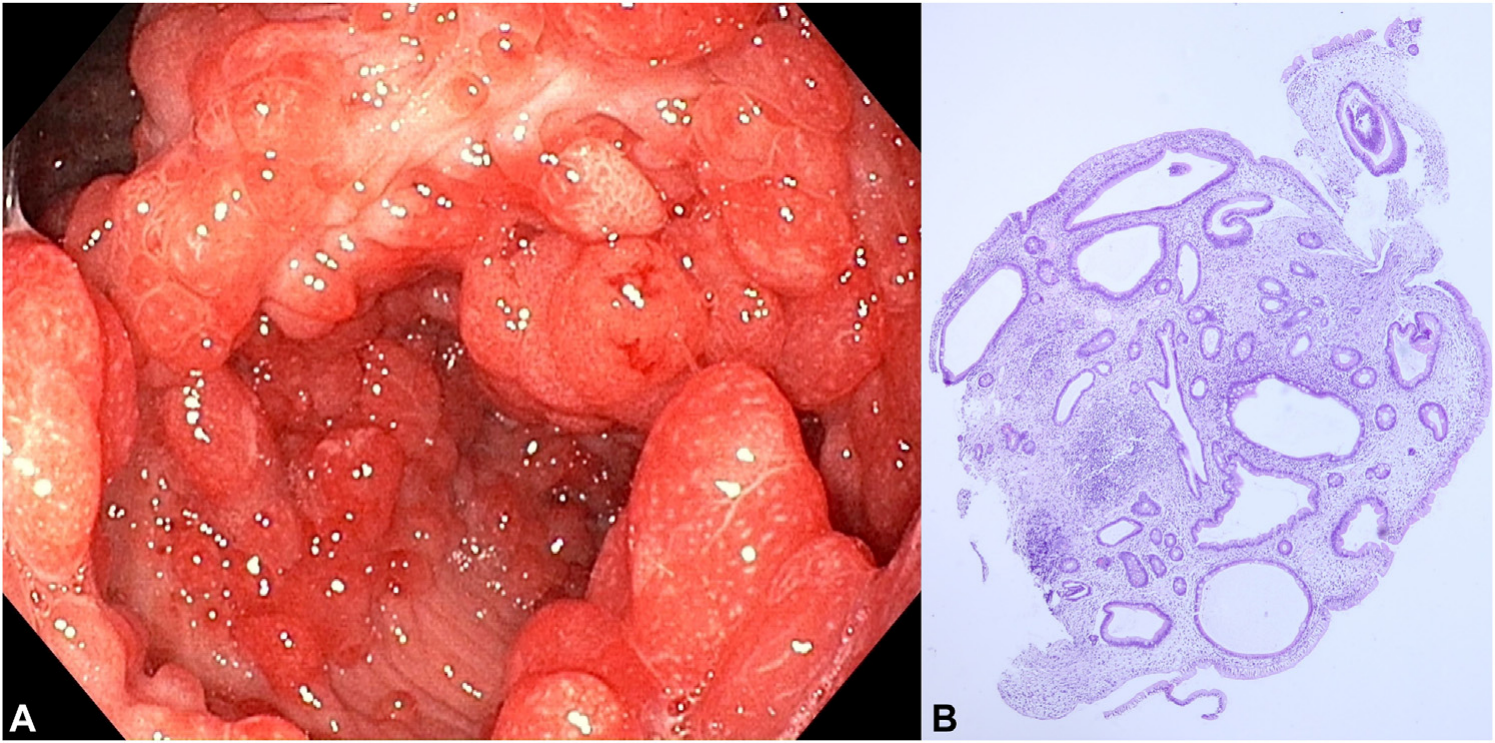
Esophagoduodenoscopy of Cronkhite-Canada syndrome. Colonic inflammatory polyps (**A**). Hematoxylin and eosin-stained histopathological findings of the polyp showed juvenile hamartomatous polyp with cystic dilation of glands and infiltration of inflammatory cells including eosinophils (**B**).

Nail clippings showed negative periodic acid-Schiff stain and potassium hydroxide preparation. The culture of nail plate revealed *Candida parapsilosis*. esophagoduodenoscopy, colonoscopy, and video capsule endoscopy demonstrated diffuse strawberry-like inflammatory polyps in the stomach, duodenum, ileum, colon, and rectum (Fig 3, *A*). Rapid urease test was not performed. Histopathological examination of the polyps revealed juvenile hamartomatous polyps with cystic dilation of glands and infiltration of inflammatory cells including eosinophils (Fig 3, *B*). IgG4 staining was not performed. A technetium-99m human serum albumin scan demonstrated protein-losing enteropathy in her cecum and ascending colon. Investigations revealed low serum albumin levels, total vitamin D and A, and normal complete blood count, liver, kidney, and thyroid function, serum iron, copper, zinc, vitamin E, B1, B2, and B6 levels.

**Fig 4:**
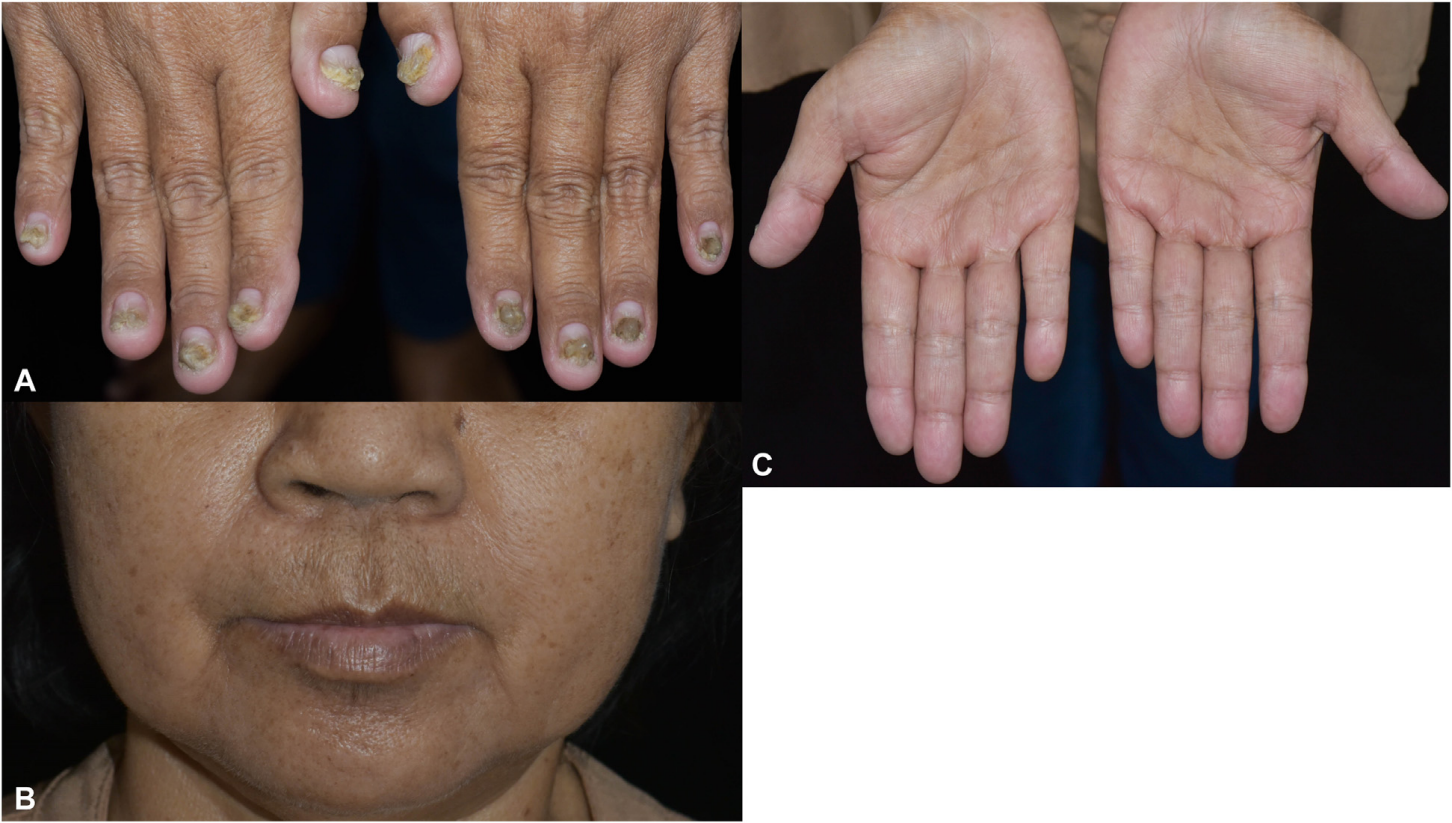
Cutaneous features improvement of Cronkhite-Canada syndrome. Proximal nails appeared normal (**A**). The *brownishmacules* perioral area (**B**) and palms (**C**) turned lighter.

Her diagnosis was CCS and protein-losing enteropathy with secondary hypoalbuminemia, vitamin A, and vitamin D deficiencies due to the distinctive histopathological result of her gastrointestinal polyps and ectodermal findings. Oral prednisolone 0.9 mg/kg/d was prescribed for 3 weeks and then tapered by 5 mg weekly. Supplementation of vitamin A at 25,000 IU per day and ergocalciferol at 20,000 IU per week was initiated. Six months after treatment, her diarrhea and nausea have improved. Her proximal nails appear normal, and her hair loss and hyperpigmentation have improved (Fig 4, *A*-*C*).

## Discussion

Onychodystrophy is the characteristic nail change associated with CCS.^6^^,^^7^ Anecdotally, it was secondary to nutritional deficiencies. Contrary to previous beliefs, recent studies suggested that chronic inflammation of the nail matrix may be a major contributor. Chuamanochan et al demonstrated hypergranulosis of the stratum granulosum and lymphocyte infiltration from a nail matrix biopsy of a patient with CCS, suggesting that nail changes in CCS resulted from systemic inflammation rather than nutritional deficiencies.^8^ However, other systemic inflammatory diseases like systemic lupus erythematosus did not manifest nail changes indicative of nail structure inflammation. Consequently, the pathological mechanism behind these nail changes has yet to be fully elucidated.

In our case, onychodystrophy and subungual hyperkeratosis were noted, representing the pathology of the nail matrix and nail bed, respectively. Vitamin A deficiency may contribute to onychodystrophy; however, hypoalbuminemia, the most common nutritional deficiency found in CCS, has never been reported as causing these nail findings.^9^ It is unlikely that nutritional deficiencies could explain nail changes.

Subungual hyperkeratosis results from nail bed inflammation and is commonly observed in psoriasis and onychomycosis. In our patient, psoriasis was ruled out due to the absence of characteristic nails, cutaneous plaques, or joint symptoms. Onychomycosis which involves all nails often occurs in an immunocompromised host. *C parapsilosis* infection usually presents with chronic paronychia, transverse grooves and ridges, and hyperkeratosis of the nail plate.^10^ Furthermore, her nails improved with systemic corticosteroid treatment. This data discount a fungal infection as a cause of her subungual hyperkeratosis. No nutritional deficiencies have yet to be linked to subungual hyperkeratosis. Thus, we believe that the subungual hyperkeratosis was associated with inflammation in the nail bed.

Our patient endured this syndrome for years before diagnosis. The prolonged systemic inflammation likely contributes to extensive involvement of her nail structures, affecting both the nail matrix and nailbed. This explains the unique nature of her nail presentation. Consequently, we propose that subungual hyperkeratosis represents a distinctive feature of CCS, particularly in cases with prolonged systemic inflammation.

The standard treatment includes systemic corticosteroids and nutritional support. A study demonstrated that treatment with prednisolone dosing at 30 to 49 mg/d and total parenteral nutrition resulted in over an 85% clinical response.^2^ Over half of the patients received systemic steroids for at least a year, with over two-thirds achieving endoscopic remission. Nail improvement occurred 3 months after starting systemic corticosteroid treatment. Our patient responded to systemic steroid treatment and vitamin A and D supplementation within 6 months. Steroid-sparing medications including cyclosporine, octreotide, and TNF-alpha inhibitors, have been described in literature and may be useful as second-line treatments.^2^

Despite a 47.3% mortality rate from gastrointestinal malignancy and malnutrition, some patients demonstrated a reversal of gastrointestinal and ectodermal symptoms when treated with systemic corticosteroids and nutritional support.^2^^,^^5^ Gastrointestinal polyp biopsies revealed no metaplasia or dysplasia, but ongoing surveillance with endoscopies is necessary due to the persistent concern of malignancy.

## Conclusion

Subungual hyperkeratosis could be considered an ectodermal feature of CCS, especially in cases with prolonged symptom duration. The diagnosis is confirmed by exclusion. We hope that this variation in clinical presentation might help us gain more insight into the pathogenesis of the disease.

## Conflicts of interest

None disclosed.
